# Complex X chromosome rearrangement associated with multiorgan autoimmunity

**DOI:** 10.1186/s13039-015-0152-5

**Published:** 2015-07-19

**Authors:** Irén Haltrich, Henriett Pikó, Horolma Pamjav, Anikó Somogyi, Antónia Völgyi, Dezső David, Artúr Beke, Zoltán Garamvölgyi, Eszter Kiss, Veronika Karcagi, György Fekete

**Affiliations:** 2nd Department of Pediatrics, Semmelweis University, Tűzoltó utca 7-9, 1094 Budapest, Hungary; Department of Molecular Genetics and Diagnostics, National Center of Public Health, Budapest, Hungary; DNA Laboratory, Institute of Forensic Medicine, Network of Forensic Science Institutes, Budapest, Hungary; 2nd Department of Medicine, Semmelweis University, Budapest, Hungary; Department of Human Genetics, Organization National Institute of Health Dr Ricardo Jorge, Lisbon, Portugal; 1st Department of Obstetrics and Gynecology, Semmelweis University, Budapest, Hungary

**Keywords:** Complex X chromosome rearrangement, Autoimmune disease, Turner syndrome, Type 1 diabetes mellitus, X inactivation disorder

## Abstract

**Background:**

Turner syndrome, a congenital condition that affects 1/2,500 births, results from absence or structural alteration of the second sex chromosome. Turner syndrome is usually associated with short stature, gonadal dysgenesis and variable dysmorphic features.

The classical 45,X karyotype accounts approximately for half of all patients, the remainder exhibit mosaicism or structural abnormalities of the X chromosome. However, complex intra-X chromosomal rearrangements involving more than three breakpoints are extremely rare.

**Results:**

We present a unique case of a novel complex X chromosome rearrangement in a young female patient presenting successively a wide range of autoimmune diseases including insulin dependent diabetes mellitus, Hashimoto’s thyroiditis, celiac disease, *anaemia perniciosa*, possible inner ear disease and severe hair loss. For the genetic evaluation, conventional cytogenetic analysis and FISH with different X specific probes were initially performed. The complexity of these results and the variety of autoimmune problems of the patient prompted us to identify the exact composition and breakpoints of the rearranged X as well as methylation status of the X chromosomes. The high resolution array-CGH (assembly GRCh37/hg19) detected single copy for the whole chromosome X short arm. Two different sized segments of Xq arm were present in three copies: one large size of 80,3 Mb from Xq11.1 to Xq27.3 region and another smaller (11,1 Mb) from Xq27.3 to Xq28 region. An 1,6 Mb Xq27.3 region of the long arm was present in two copies. Southern blot analysis identified a skewed X inactivation with ≈ 70:30 % ratios of methylated/unmethylated fragments. The G-band and FISH patterns of the rearranged X suggested the aspect of a restructured i(Xq) chromosome which was shattered and fortuitously repaired. The X-STR genotype analysis of the family detected that the patient inherited intact maternal X chromosome and a rearranged paternal X chromosome.

The multiple Xq breakages and fusions as well as inverted duplication would have been expected to cause a severe Turner phenotype. However, the patient lacks many of the classic somatic features of Turner syndrome, instead she presented multiorgan autoimmune diseases.

**Conclusions:**

The clinical data of the presented patient suggest that fragmentation of the i(Xq) chromosome elevates the risk of autoimmune diseases.

## Background

Turner syndrome (TS) is a known genetic disorder associated with complete or partial absence of one of the sex chromosomes (the X or Y chromosome) affecting about one out of 2500 liveborn girls. The most common phenotypic features of TS patients are short stature and gonadal dysgenesis, they are at significantly increased risk of different congenital malformations and prone to develop gradually autoimmune disorders.

The isochromosome X [i(X)(q10)], which consists of two copies of the long arm with missing short arm is the most common structural rearrangement, occurring in about 20 % of TS cases. By the currently applied techniques complex X chromosome rearrangements having three or more breakpoints are rarely seen in constitutional karyotype, even rarer are rearrangements occurring within a single chromosome. To date, only seven previous reports of complex X chromosomal rearrangement have been described [1–7].

Here we report a novel complex X chromosome rearrangement in a young TS patient with type 1 diabetes mellitus (DM) and severe other endocrine complications. We delineated the highly rearranged derivative X chromosome by G-banding and FISH analysis as well as array comparative genome hybridization (CGH; assembly GRCh37/hg19). The presented X chromosome rearrangement can bring insight into the possible genetic mechanisms responsible for X-linked autoimmune disorder.

## Case presentation

The patient was born from an uncomplicated pregnancy, on term per *vias naturales* with 2750 grams. She was the first child of a 20-year-old mother and a 23-year-old father. The mother was a joiner, the father dealt forest yields, and he drank excessively by his late adolescence. He lost his sight because of alcohol use disorder and repeatedly attempted suicide. He died premature, at age of 24 years because of alcohol induced central nervous system damage. The father’s parents are farmers; they are healthy, without any family history of alcoholism. The mother’s father worked in a quarry and the mother was housewife. Apart from the female cousin suffering from type 1 diabetes mellitus (DM), the family history is unremarkable. Genetic and autoimmune disorders, mental retardation have never occurred. The patient’s mother had one miscarriage and one healthy son with a second husband.

At newborn age the patient presented no stigmata of Turner syndrome. At the age of 17 years she was referred to cytogenetic laboratory due to her primary amenorrhoea. Standard karyotype analysis had shown a complex rearrangement of the one X chromosome.

On examination, her height was 152 cm, her weight was 50 kg. Her short stature wasn’t conspicuous because parental grandparents, her mother’s sibling and several cousins were at the height around of 155 cm. The proband’s predicted final height is 158, 5 cm, currently she is 24 years old and 154 cm in height (<3^rd^ percentile, growth rate - 2 SD).

She had spontaneous breast development (Tanner stage III-IV) but sparse axillary and pubic hair (Tanner stage II). Laboratory studies revealed the following values: FSH level: 77.83 IU/L (normal women: >40 IU/l), LH level: 24.20 IU/L (normal women: >21 IU/l), E2 5 pg/ml (normal women: up to 375 pg/ml), confirming primary ovarian insufficiency (POF). Receiving estradiol therapy her secondary sexual characteristics have developed and her menstruation was normal. Since ten years of age, she has been suffering from hearing loss on both ears which was resolved by a hearing aid. She had recurrent respiratory infections, she also suffered from pyelonephritis. At the age of 13 years after tooth extraction mandibular osteomyelitis developed, which was successfully treated with antibiotic therapy. At the age of 15 years she was diagnosed with type 1 DM and insulin substitutive treatment was started. In the same year *anaemia perniciosa* was diagnosed as the underlying cause of her anaemia. She received vitamin B12 regularly. Some month later levothyroxine therapy was introduced due to the diagnosis of Hashimoto thyroiditis with clinical hypothyroidism. Her thyroid function was normal under the therapy (TSH: 3.17 mIU/l). At the same time she presented symptoms of coeliac disease. The immunhistochemistry examination of gastric mucosa revealed an increased level of chromogranin A. The gastroscopy examination explored neuroendocrine cell hyperplasy in the antral and corpus regions. When dydrogesterone was added to her hormone replacement therapy, she suffered from severe hyperglycaemia and ketoacidosis and intensive care was necessary. The estradiol dydrogesterone therapy was temporarily stopped due to insufficient carbohydrate metabolism. Initially, for two weeks an insulin pump was introduced to regulate her carbohydrate metabolism. Due to her inability to learn the use of the insulin pump (memory deficit) regular insulin therapy was continued. Without the hormone replacement therapy the patient had an amenorrhoea. Therefore, hormone replacement therapy was newly added and well tolerated without any ketoacidotic periods until today. At the age of 23 years gynaecological ultrasound examination showed that the myometrium of the uterus is normal (size of the uterus: PF: 32 mm, AP: 16 mm; endometrium: 4 mm.). Both ovaries were smaller as compared with the size of normal reproductive-aged women (the right ovary with 9x6 mm, without any follicles; the left ovary with 10x7mm, without follicles) and suspect for streak gonads and infertility. No free fluid in Douglas was detected. The patient’s other current medical problems include osteoporosis, eczema and severe hair loss. Her behaviour is conventional and social; a mild intellectual failure could be observed.

## Methods

### Cytogenetic analysis

Chromosome analysis was performed on phytohemagglutinin stimulated peripheral blood lymphocyte cultures on metaphase cells with trypsin and Wright Giemsa stain. Fluorescence *in Situ* Hybridization (FISH) analysis was carried out on methanol/acetic acid-fixed suspensions. Slides preparation for FISH was made according to standard techniques. X and Y centromere (CEP X and Y) specific probes as well as X, Y painting probe, SRY specific probes were used for evaluation of exact genotype and to detect chromosome X copy number. CEP X specific FISH probe was investigated also for buccal and urine mucosa interphase cells of the patient in order to reveal the possible tissue specific X chromosome mosaicism. To determine the composition of rearranged X chromosome, additional hybridizations were effectuated with X p arm red, Xq arm green, SHOX, XIST (Cytocell, United Kingdom) as well as subtelomere Xq/Yq specific (ToTelVysion, Abbott, Germany) probes. Karyotypes and FISH results were described according to the International System for Human Cytogenetic Nomenclature (ISCN 2013).

### Southern blot analysis

For X chromosome methylation status determination FRAXA analysis was initiated. Genomic DNA from the patient was isolated from peripheral lymphocytes by the simple salting-out procedure. DNA was subjected to restriction enzyme digestion with EcoRI and the methylation sensitive enzyme EagI followed by Southern blot analysis and hybridization using the DNA probe StB12.3 [8]. Methylated normal alleles are not cut by methylation sensitive enzymes and give rise to the normal 5.2 Kb EcoRI fragment. In unaffected females, two bands are visible: a 2.8 Kb fragment corresponding to the unmethylated/active X and a 5.2 kb allele representing the methylated/inactive X chromosome and the signal strength of both fragments are more or less equal. In case of FRAXA premutations or full mutations, methylated and unmethylated CGG expansions are indicated by the presence of bands and/or smears above 5.2 Kb and 2.8 Kb, respectively.

### Repeat primed PCR analysis

Repeat primed (RP) PCRs were performed on 20-40 ng DNA according the supplied protocol from Asuragene. This protocol supports two different PCR formats, gene specific *FMR1* PCR and CGG Repeat Primed (RP) PCR. The gene specific PCR contains two primers outside the CGG repeat in *FMR1* gene, and the CGG Repeat Primed PCR contains 3 primers: two outside and one inside the CGG repeat.

### Array comparative genomic hybridization

To ascertain the segmental composition of the rearranged X chromosome, a high resolution genomic scan using ISCA plus design array of NimbleGene-Roche containing 1.4 M probes per subarray (assembly GRCh37/hg19) was performed in the DNA samples of the patient. This CGX microarray provides a mean average resolution of approximately 15-20 Kb. Array CGH analysis was performed according to the manufacturer’s protocol. The CGH protocol involves independent labelling of the patient (test DNA) and the reference genomic DNA (Human Genomic DNA, Promega Madison, WI U.S.A.) with Cy3 and Cy5 dyes using a NimbleGene Dual-Color DNA Labelling Kit (Roche-NimbleGen Inc.). Co-hybridization of these DNAs to a NimbleGene CGH array was performed for 72 h at 42 °C. The subarrays were scanned on NimbleGene MS 200 microarray scanner and data were extracted and analysed using NimbleScan, SignalMap and Deva 1.1 softwares (Roche NimbleGene Inc.). DNA CNVs were mentioned as gain or loss in a linear ratio, and the length of the variation was given in megabase (Mb) and base pair (bp) respectively.

### Identification of the rearranged X chromosome origin

In order to identify the origin of the rearranged X chromosome, we have tested 12 X- chromosomal STR (Short Tandem Repeat) markers on the entire length of the X chromosome according to the manufacturer’s instructions. The Investigator Argus X-12 Kit (Qiagen, Hilden, Germany) is a multiplex PRC kit for determination of 12 X-STR loci simultaneously. The kit contains primers for Amelogenin for gender determination, DXS7132, DXS7423, DXS8378, DXS10074, DXS10079, DXS10101, DXS10103, DXS10134, DXS10135, DXS10146, DXS10148, and HPRTB loci. The markers are clustered into 4 linkage groups (3 markers per group), and each set of 3 markers is closely linked and handled by a haplotype for genotyping. These 12 X-STR loci are located in four linkage groups as followings: on Xp22: DXS8378 – DXS10135 – DXS10148; on Xq11.2-Xq12: DXS7132 – DXS10074 – DXS10079; on Xq26: HPRTB – DXS10101 – DXS10103 and on Xq28: DXS7423 – DXS10134 – DXS10146 loci. The PCR products were analyzed by capillary electrophoresis using an ABI 3130 Genetic Analyzer (Life Technologies, Foster City, CA). The fragment sizes and allele designations were determined by using the GeneMapper ID 3.2.1 (Life Technologies, Foster City, CA).

### WAIS-IV intelligence test

The Wechsler Adult Intelligence Scale, Fourth Edition (WAIS-IV) [9] is a comprehensive clinical instrument for assessing intelligence of adults between the ages of 16–90 years. The test is composed of 15 core and supplemental subtests which contribute to a composite score that represents general intellectual ability (full scale IQ, FSIQ) and scores in indices of specific cognitive areas: Verbal Comprehension Index (VCI), Perceptual Reasoning Index (PRI), Working Memory Index (WMI), and Processing Speed Index (PSI).

## Results

**Fig. 1:**
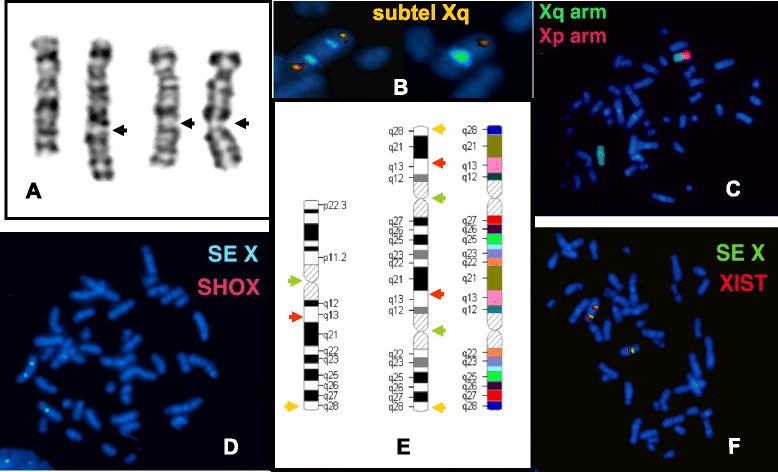
The G-banding and FISH analysis of the rearranged and normal X chromosome. The G-band pattern of the normal and rearranged X chromosome showing multiple appearance from different metaphases of the rearranged X chromosome depending on the state of condensation; the black arrows indicating the localization of the second centromeres of the rearranged X (**a**), cohybridization of X centromere (aqua) and subtel Xq probes (red-green) showing the two centromeres of derivative X chromosome both extremities painted with Xq subtelomere specific probe as well as the normal pattern of the homologue X (**b**); chromosome paint probe for the short arm (red) and the long arm (green) showing the derivative X composed only from Xq arm (**c**); SHOX region could be identified only on normal X chromosome (**d**); The wild-type and rearranged X chromosome ideograms with arrows showing the position of the used FISH probes: subtelomere Xq (yellow), X centromere (green), XIST (red); colored ideogram of the rearranged X indicating the sequential order of bands suggested by G-band and FISH analysis (**e**); duplicated XIST probe in red, and the centromeric marker in green (**f**)

The proband was referred to the cytogenetic laboratory at 17 years of age. The G-banded analysis revealed two X chromosomes in all the 25 metaphases examined: one normal and the other one rearranged with altered banding pattern composed of an undersized short arm and an elongated long arm (Fig. 1a).

Cohybridization of X centromere and subtel Xq probes showed a normal X chromosome with one centromere and one subtel Xq signal and a derivative dicentric X chromosome with both arms ending with subtelomere Xq (Fig. 1b). The arm painting probes revealed a normal p and q pattern X chromosome and a derivative X fully highlighted with X q arm specific paint probe (Fig. 1c). Hybridization with X centromere and *SHOX* specific probe did not yield any *SHOX* gene signal on the dicentric X chromosome (Fig. 1d). The FISH examination with the X centromere/XIST specific probe detected a normal monocentric X with one XIST signal and a dicentric X with two XIST signals (Fig. 1f). X centromere probe hybridization of urine and buccal mucosa cells showed the same pattern in all 100-100 analysed interphase as lymphocytes indicating the absence of mosaicism. The proband’s mother and half-brother have normal karyotype. The relatives of the patient’s father are not accessible.

**Fig. 2:**
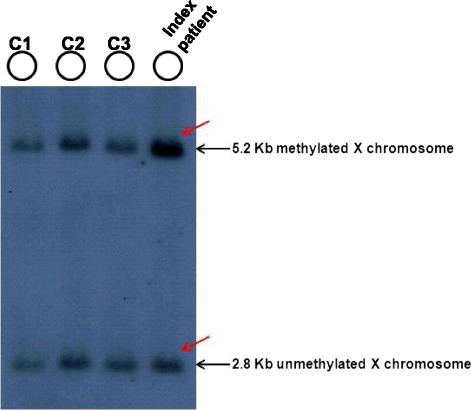
Southern blot analysis of the patient and of control females. EcoRI and EagI double digested DNA samples hybridized with radioactive-labelled Stb12.3 probe were used for Southern blot analysis. Black arrows indicated the 2.8 kb unmethylated and the 5.2 kb methylated fragments. For the index patient (red arrow) we can define two copies of 5.2 kb methylated fragments and one copy of 2.8 kb unmethylated fragment. For all controls we identified two normal alleles (2,8 kb unmethylated and 5.2 kb methylated) characteristic for two normal X chromosomes

**Fig. 3:**
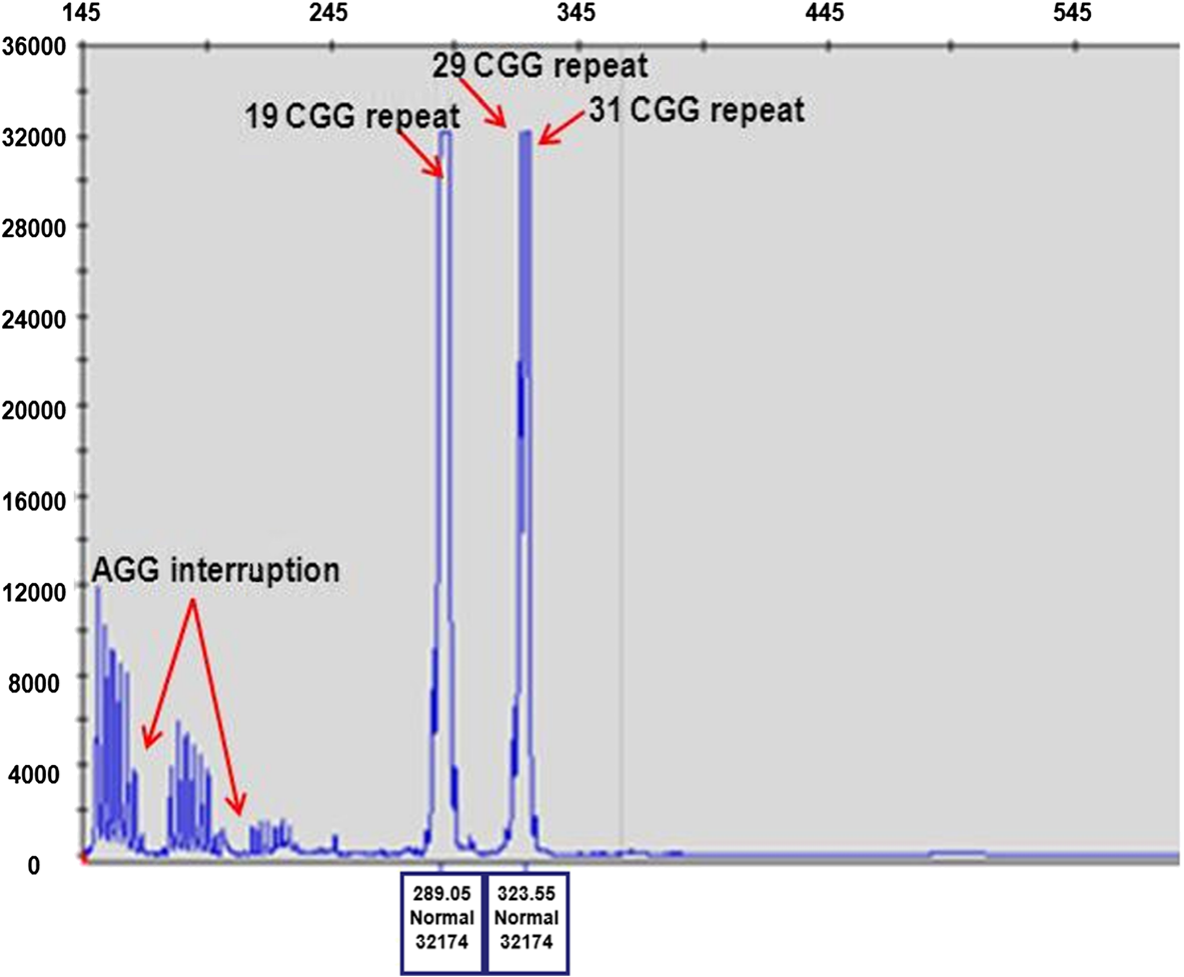
Repeat-Primed PCR analysis. Repeat-primed PCR analysis revealed three peaks corresponding to a 19, 29 and 31CGG repeat representing three FMR1 gene alleles (red arrow). The method is also suitable for detection of AGG sequences interrupting the CGG repeats. In the index patient we determined two interruptions (red arrow). The AGG repeats stabilize the CGG repeats containing sequences

In order to compare the X-chromosome inactivation (XCI) ratios between the two X-chromosome of the patient, we performed Southern blot analysis (Fig. 2) which revealed the 5.2 Kb methylated fragment with 2-times intensity compared to the 2.8 Kb unmethylated Xq27.3 fragment (approx. with 70:30 % ratio), assuming the presence of three FMR1 alleles with a skewed X inactivation. In the contrary, for all controls we identified two X chromosome normal alleles (with a 50:50 % value of the 2,8 kb unmethylated and the 5.2 kb methylated fragments). In order to ascertain the presence of three Xq arms we performed repeat-primed PCR analysis which revealed three peaks corresponding to a 19, 29 and 31CGG repeats, proving the existence of three *FMR1* genes (Fig. 3).

**Fig. 4:**
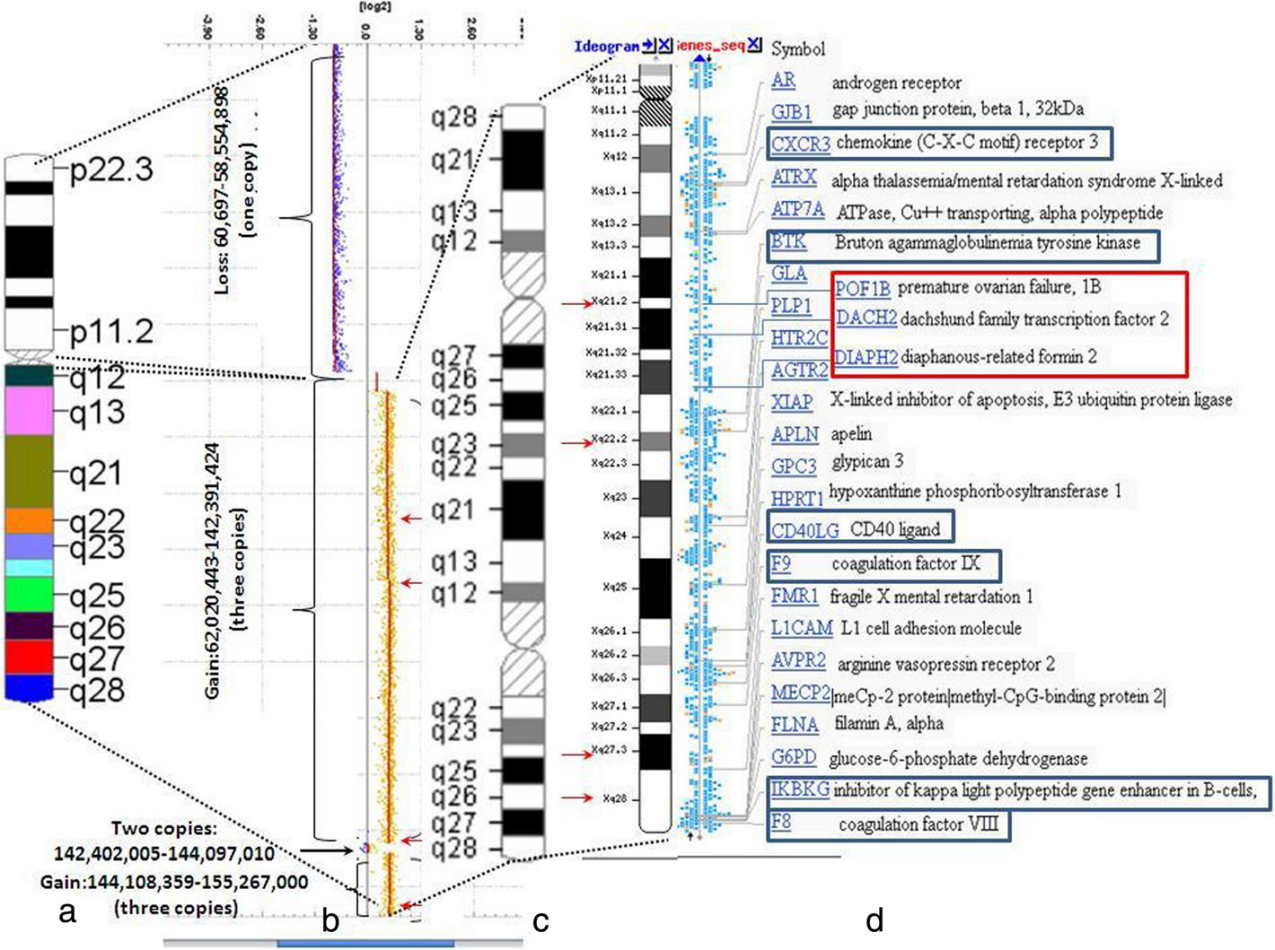
Detailed analysis of the rearranged X chromosome using NimbleGene Array (assembly GRCh37/hg19). **a** The ideogram delineates genomic regions with the cytogenetic bands on normal X chromosome. **b** Panel “b” reveals the result of the array-CGH genotype. The CGX ISCA plus array showed a 58.494 Mb loss (ChrXp22.33-p11.; one copy) and an 80.370 Mb three copies gain (ChrXq11.1-q27.3). Furthermore, there is a 1.6 Mb normal two copies region (ChrXq27.3) and an 11.1 Mb three copies gain (ChrXq27.3-q28). The red arrows represent the breakpoints. **c** Panel “c” indicates genomic regions with cytogenetic bands on the complex rearranged X chromosome of the patient. **d** Panel “d” represents genes and positions which are affected in this patient. The blue rectangle indicates those genes which can be associated with immunodeficiency and the red rectangle represent the genes involved in POF phenotype. The red arrows represent the breakpoints

Array-CGH (NimbleGene Array CGX 1.4 M; assembly GRCh37/hg19) detected single copy for the whole chromosome X short arm (involving Xp11.1-22.33 region and 60,697 - 58,554,898 bp). Chromosome Xq27.3 region of the long arm flanked by 142,402,005 – 144,097,010 bp was present in two copies. Two segments with different sizes of Xq arm were present in three copies: one large of 80,3 Mb ranging from Xq11.1 to Xq27.3 comprising of 62,020,443 - 142,391,424 bp and another smaller one (11,1 Mb) from Xq27.3 to Xq28 region flanked by 144,108,359-155,267,000 bp (Fig. 4). The G-banding pattern suggests that the rearranged X results from several breakages succeeded by segmental inverted duplication, deletion and reunion of different Xq segments. On the bases of array finding the breakpoint could be localized on the Xq21.2, Xq22.2, Xq27.3 and Xq27.3-Xq28 regions (Fig. 4). These findings suggest that the short arm of the abnormal X chromosome was totally lost and the remaining part was likely a structurally reorganized isochromosome of the long arm [i(Xq)] (Fig. 1e). Based on 200 interphase cells X centromere analysis there was no evidence of mosaicism. Y chromosome whole painting and SRY specific probes hybridization were negatives. The patient’s mother has normal karyotype; her father was already deceased.

**Fig. 5:**
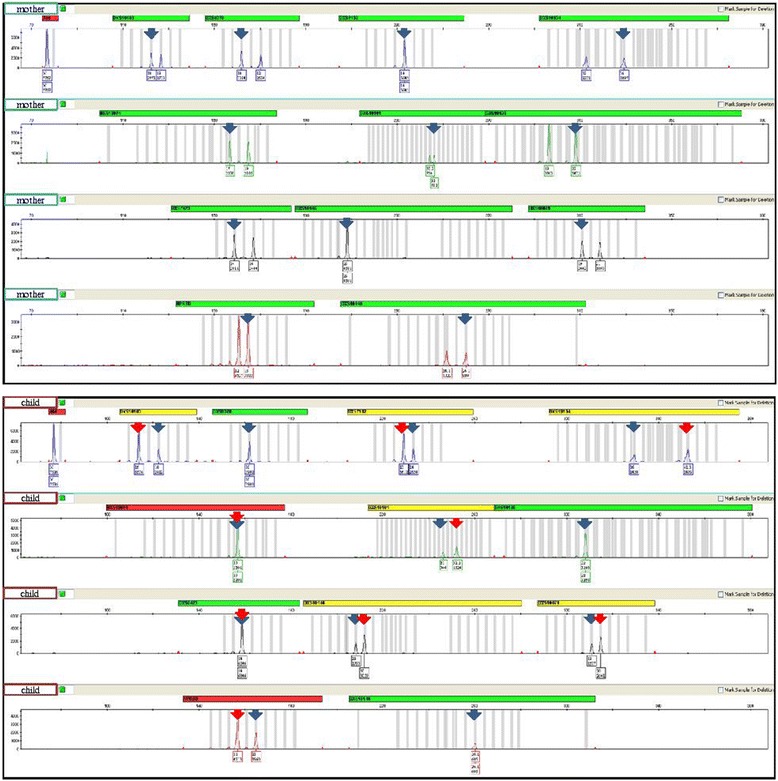
X chromosomal genotypes of the mother and the patient. The Investigator Argus X-12 kit contains 12 X-chromosomal STR loci located on both arms of the X chromosome. Linkage group 1 (Xp22.31) contains DXS10148, DXS10135 and DXS8378, Linkage group 2 (Xq11.2-12) contains DXS7132, DXS10079 and DXS10074, Linkage group 3 (Xq26.2) contains DXS10103, HPRTB and DXS10101, and Linkage group 4 contains DXS10146, DXS10134 and DXS7423 loci, and they are inherited as haplotypes. Blue arrows point to the maternal and red arrows point to the paternal alleles. The first number under a peak designates the name of the allele, and the second number the height of the peak in RFU (Relative Fluorescence Unit). The peak heights of the mother’s alleles within a locus are very similar indicating equal template concentration during PCR. Contrary, the peak heights of the patient’s heterozygote alleles show discrepancy of the template DNA (approximately doubled paternal allele intensities). From the X-STR genotypes it can be concluded that the patient inherited intact maternal X chromosome and a rearranged paternal X chromosome with no short arm and two long arms

Based on the X-STR studies we have identified the origin of the rearranged X chromosome: the alleles inherited from the mother to the patient are highlighted in red colour and the other ones on the second X chromosome are highlighted in black (Table 1). Alleles originated from the father to the patient are highlighted in blue (Fig. 5, red arrows). As seen in the linked loci DXS10148-DXS10135-DXS8378 and their alleles (26.1, 22 and 10 in the patient) on Xp22.31, these are in red colour indicating only one copy of X chromosome short arm (see the lower peak heights and blue arrows in Fig. 5 as well) and are inherited from the mother. It is interesting to see the genotype of the maternal half sibling of the patient that the boy inherited a recombinant X chromosome from the mother showing that a recombination occurred between Linkage group 1 (Xp22.31) and Linkage group 2 (Xq11.2-12) between two the maternal X chromosomes (his electropherogram is not included in Fig. 5). In other words, the same Xq arm of the mother in the case of the patient (red alleles) and the other Xp arm of the maternal X chromosome (black alleles 24.1, 19 and 12) are transmitted to the half sibling. Furthermore, it can be seen that a recombination never occurs within a closely linked loci as we see 3 black alleles on Xp22.31 as described above in the half sibling.

**Table 1:** X-chromosomal genotypes of the affected family

The administered WAIS–IV psychological test identified a borderline score for general intellectual ability (FSIQ: 70, percentage rank: 2). The verbal concept formation, comprehension and expression as well as word knowledge, were significantly below average with VCI: 58 and 0,3 percentage rank. The perceptual reasoning ability, visual perception and organization, spatial visualization and manipulation, and the ability to analyze and synthesize abstract visual stimuli were at lower average than her age correspondent average score (PRI: 79, percentage rank: 8). She achieved a borderline score (WMI: 80; percentage rank: 9) in the field of working and short-term memory, memory span, attention and concentration. Her measured short –term visual memory and perception was also borderline (PSI: 84; percentage rank: 2).

## Discussion

Here we report the case of a young female patient with a unique X chromosome complex rearrangement and exhibiting sequentially a wide range of autoimmune diseases. Initially, G-banding and X-specific FISH examination were performed, but the complexity of these results and the varied clinical findings prompted us to carry out the high resolution array-CGH as well. However, the rearranged X of the presented case was missing the typical two “mirror – image” specific for i(Xq), the Xq arms were present in three copies, except for 1,6 Mb from band q27.3 which could be considered as breakpoint related loss. On the other hand, the rearranged X - harbouring two centromeres, inversions and several breakpoints - was much more complex as a common i(Xq) chromosome. In order to determine the parental origin of the rearranged X, we analyzed the X specific STR markers of the patient, her mother and half brother. Males carry one X-chromosome, thus they transmit their X chromosome to daughters as haplotypes. Females carry two X chromosomes which are liable to recombination during meiosis and transmit one of their X chromosomes to children regardless of genders. Population genetic study for 12 X-STR loci and X chromosomal recombination study in three-generation families in Hungary, were reported before [10, 11]. Due to extremely polymorphic X-STR loci and low haplotype frequencies of the linked loci, we were able to solve this question.

In the present study we tested 12 X-STRs on the entire length of X chromosome within 4 linkage groups to identify the origin of the affected X chromosome in the patient. Based on the study we have clearly identified not only the origin of the affected and non-affected X chromosome but also gained information on the copy number of the X chromosomal short and long arms even if the father was not available for testing. As we described in Results (Table 1 and Fig. 5) on the basis of the peak heights of the mother’s and patient’s alleles within a locus and the X-STR genotypes detected it can be concluded that the patient inherited an intact maternal X chromosome and a rearranged paternal X chromosome with no short arm and two long arms.

Recent studies [12] elucidated that most of the isodicentric X chromosomes [idic(Xq)] result from non-allelic homologous recombination between palindromic low copy repeats and highly homologous palindromic LINE elements. We could speculate that during the paternal meiosis the preformed i(Xq) chromosome could have undergone several breakage due to a catastrophic mutagen effect (severe alcohol abuse?) and this event was followed by wrong reunion route. The distal position of the two centromeres and the location of the XIST gene signals as well as the banding pattern suggest that in our case the dicentric X chromosome was initiated by centromere misdivision, multiple double-strand breakage, inversion of centromeric-Xq27.3 segment followed by a fortuitously recombination process (Fig. 1e). The fact that other tissues as urine or buccal epithelial cells did not show any signs of mosaicism suggests that this aberrated X chromosome was generated at early stage of meiosis during spermatogenesis.

Cytogenetically idic(Xq) chromosome cases had breakpoints in euchromatic sequences within a 7 Mb region of proximal Xp arm and monocentric i(Xq) within centromeric heterochromatin. On the bases of the array-CGH results the breakpoint in our case was localized to the DXZ1 array junction which marks the beginning of the functional centromere and corresponds to the known monocentric i(Xq) breakpoint [12].

To date, only seven previous reports of complex X chromosomal rearrangements have been described [1–7]. To the best of our knowledge this is the first complex X chromosome rearrangement reported case associated with type 1 DM and other four autoimmune diseases.

Loss of genetic material from one of the sex chromosomes causes the distinct clinical phenotype of TS: reduced adult height, gonadal dysgenesis, variable dysmorphic features. (low posterior hairline, webbed neck, broad chest, short fourth metacarpals). Congenital heart defects, structural renal anomalies, hearing concerns, autoimmune diseases and cognitive deficits with particular difficulties in visuo-spatial and memory areas are common in TS patients.

The presented patient in our study has lack of the typical Turner phenotype. She is 154 cm but her short height is not obvious because she has close relatives with similar height; dysmorphic features are absent, she is rather an attractive young woman. Turner diagnosis was suggested by POF, the concurrent health concerns such as severe hearing loss, type 1 DM, hypothyreosis, *anaemia perniciosa,* celiac disease; severe hair loss (*alopecia areata*) and repeated severe infections were the main health problems.

TS patients usually have a typical neurocognitive profile characterized by average to low-average full-scale intelligence quotient (IQ). Intellectual disability is defined as having an IQ score below 70; in our case the administered WAIS-IV intelligence test identified a borderline score for the full-scale IQ (FSIQ: 70) and borderline score for the three supplemental subsets indices (PRI, WMI, PSI); furthermore her “verbal intelligence” was well below average (VCI: 58).

POF is defined as a primary ovarian defect characterized by amenorrhea, low levels of gonadal and high levels of gonadotropin hormones. Deletions on the X chromosome reduce both fertility and reproductive lifespan. Two loci on Xq have been postulated as the critical regions for POF: POF1 on Xq27.3-q28 (OMIM #311360) and POF2 (*POF2B* on Xq21.1q21.2; OMIM #300604 and *POF2A* on Xq21.33; OMIM # 300511) [13, 14].

The breakpoints of the rearranged X chromosome presented were adjacent to the POF critical regions. These findings suggest that the breakpoint related lesions could be responsible for the patient’s POF. We assume that at the Xq21.2 breakpoint two genes, *POF1B* and *DACH2* could be affected. These genes are located 700 kb apart, proximal to the centromere; *POF1B* is expressed during early ovarian development and *DACH2* during early ovarian follicular differentiation process. The interruption of both genes by X-autosomal translocations was described in many patients with POF and Xq21.2 has been postulated as critical region for infertility and reproductive lifespan. Furthermore, we presume that the other gene in question in the Xq21.33 breakpoint is *DIAPH2* [NM_007309]. The product of this gene belongs to the diaphanous subfamily of the formin homology family of proteins. This gene may play a role in the development and normal function of the ovaries. Defects in this gene have been linked also to premature ovarian failure 2 (POF2).

The presented patient suffered from severe bilateral hearing loss which needed a hearing aid from six year of age. Otologic disease is an important characteristic in Turner syndrome [15]. The associated hearing impairment has been described as both conductive and sensorineural, signifying both middle and inner ear involvement. Recent studies suggested that there is a correlation between frequency and severity of hearing problems and karyotype: a higher occurrence of hearing problems and an age-related deterioration of hearing thresholds is expected in children with X monosomy or i(Xq) compared to those with a mosaicism or other structural anomaly [16]. Based on these findings, the X chromosome p arm loss may have some influence on the development of hearing loss phenotype [17].

Several studies reported an increased risk of autoimmune diseases among women with Turner syndrome, most notably Hashimoto’s thyroiditis, diabetes mellitus, celiac disease, ulcerative colitis, Crohn’s disease, psoriasis, idiopathic thrombocytopenic purpura, vitiligo and juvenile rheumatoid arthritis [18–22]. Recent studies describe a 4 to11-fold increased risk of -usually mild- diabetes in Turner syndrome patients compared to normal females, with typically age of onset in the 30s or 40s. They explain this high prevalence with the impaired glucose homeostasis in TS patients and with their higher glycemic response to oral glucose load and significantly lower insulin secretory response [19]. We need to point out that despite of Danish registry study [23] which found an 11-fold increase in type 1 DM among TS patients, TS patients seem to have an increased incidence of adult-onset, type 2 DM related to obesity and /or insulin resistance. Based on the literature search the association of TS and type 1 DM is very rare, documented with only 1-2 case reports per European countries [21, 24–26]. In the presented patient the type 1 DM was diagnosed at the age of 13 years, was complicated with severe ketoacidosis episodes and treatment difficulties.

The increased autoimmune disease risk has been suggested to be due to haploinsufficiency of genes on the X chromosome [27]. Women with Turner syndrome resemble in number of X chromosome to the men’s karyotype. Jorgensen et al. [20] demonstrated that in Turner syndrome patients the standardized incidence ratios for autoimmune diseases with male predominance are most pronounced, more than twice higher than female predominant autoimmune diseases.

The X chromosome contains the largest number of immune-related genes of the human genome. The X-linked genes dosage is critical for maintenance or the loss of the immune tolerance. Immune related cells cannot be tolerated by self-antigens encoded by X chromosome, thus triggering an autoimmune response in target tissues [28].

Regarding the putative influence of the type of X chromosome rearrangement on clinical features, some studies demonstrated a stronger association between autoimmune diseases and trisomy for Xq [19, 22]. Jorgensen et al. [20] have been identified 3-fold increased risk for female- and 5-fold respectively for male–predominant autoimmune diseases in women with an i(Xq) karyotype. Particularly strong association was found between i(X) karyotype with ulcerative colitis, which was detected also in the presented patient. Hamza et al. [22] reported a higher prevalence of autoimmune thyroiditis in Turner patients with i(Xq) than with 45,X karyotype. Bakalov et al. [19] demonstrated that haploinsufficiency for Xp causes an increased risk for diabetes, and that the rate of DM is significantly higher (~43 %) in the i(Xq) patients. Comparing gene expression profile of 45,X and 46,X,i(Xq), they explained this higher risk by overexpression of Xq genes that may provide a link to a large number of autosomal genes involved in pancreatic islets and B-cell function. The Xq genes transcripts result an increased IGF2, CRP and GAD level which sustain a proinflammatory state and low-grade chronic autoimmunity in individuals [19, 29]. Current studies have been indicating a strong association of X –linked genes with autoimmune diseases risk and also the evidence of sex-specific effect of each of these genes in the same disease. Several of these genes have an immune-related function or interact with autosomal genes in immune-related pathways or are involved in regulation of apoptosis with role in autoimmune diseases [28, 30].

Another aspect of the idic(Xq) chromosome is the possible perturbation of the random X inactivation through which the gene dosage between male (XY) and female (XX) sexes are balanced. Inactive X chromosome is characterized by XIST RNA coating, condensation of chromatin, methylation of CpG islands, post-translational modifications of histones. We determined the methylation status of the polymorphic FMR1 gene locus and we found unequal methylated and unmethylated fragments sizes which refer to a skewed X chromosome inactivation. Skewed X chromosome inactivations have been described in females with major X chromosome rearrangements [31]. The detected methylation shift could result in our case a local escape from inactivation of genes which contributed also to the abnormal phenotype of the patient.

## Conclusions

In conclusion, the clinical data of the presented patient suggest that fragmentation of the i(Xq) chromosome compounds the risk of autoimmune diseases. The skewed X inactivation and perturbation of *XIST*-mediated silencing by the double XIST region of complex rearranged X chromosome could be an important contributing mechanism to autoimmune disorders of the patient. Another factor such as altered gene expression due to their disruption or proximity to the breakpoints, transformed position effect of genes caused by inversions and deletions could also contribute to development of multiorgan autoimmune syndrome of the patient. Our case suggests that array-based analysis and X-inactivation studies might be useful complementary clinical investigations to reveal the aetiology of primary amenorrhea and autoimmune diseases.

## Consent

Written informed consent was obtained from the patient for publication of this case report, and accompanying images. A copy of the written consent is avaible by the Editor-in –chief of this journal.

## Abbreviations

Bp: Base pair(s)
CGH: Comparative genomic hybridisation
*DACH2*: Homo sapiens dachshund homolog 2 transcript variant 3 mRNA
*DIAPH2*: Homo sapiens diaphanous-related formin 2 transcript variant 156, mRNA
DM: Diabetes mellitus
*FMR1* and *FMR2*: Fragile X mental retardation 1 and 2 genes
FRAXA: Fragile Site, Folic Acid Type, Rare, Fra(X)(q27.3) A
idic(Xq): Isodicentric chromosome of the long arm X
i(Xq): Isochromosome of the long arm X
LG: Linkage Group
Mb: Megabase(s)
POF: Premature ovarian failure
*POF1B*: *Homo sapiens* premature ovarian failure1B mRNA
RP PCR: Repeat primed polymerase chain reaction
*SHOX*: Homo sapiens short stature homeobox transcript variant 1
*SRY*: Sex Determining Region Y
STR: Short Tandem Repeat
TS: Turner syndrome
WCP: Whole chromosome painting probe
*XIST*: Homo sapiens X inactive specific transcript non-coding RNA
